# Nurse anaesthetist students' experiences of patient dignity in perioperative practice—a hermeneutic study

**DOI:** 10.1002/nop2.110

**Published:** 2017-11-30

**Authors:** Berit T. Valeberg, Ingrid Liodden, Bergsvein Grimsmo, Lillemor Lindwall

**Affiliations:** ^1^ Department of Nursing and Health promotion Oslo and Akershus University College of Applied Sciences Oslo Norway; ^2^ University College of Southeast Norway Notodden Norway; ^3^ Department of Health sciences Karlstad University Karlstad Sweden

**Keywords:** caring science, critical incidents, hermeneutic, human dignity, nurse anaesthetist student, perioperative practice

## Abstract

**Aim:**

The aim of the study was to describe how nurse anaesthetist students experienced patient dignity in perioperative practice.

**Design:**

A hermeneutical design and the critical incident technique were used to obtain experiences from practice.

**Method:**

In the Autumn of 2015, after participating in a mandatory lecture on ethics, 23 nurse anaesthetist students reported their experiences and interpretation concerning violation and preservation of patients' dignity in the operating theatre. The text, which was a compilation of descriptions of 35 incidents, was analysed by using hermeneutical text interpretation.

**Findings:**

The text revealed three main themes preserving patients' dignity: allocating time to the patient, inviting the patient to participate and shielding the patient's body. Furthermore, three main themes of dignity violation were identified: alienation, backbiting and violation of intimate sphere.

**Conclusion:**

Discussion and reflection based on the personal experience of the students during their practice are ways to strengthen ethical awareness and promote an ethical and dignified caring culture.

## 1 INTRODUCTION

Perioperative nursing care, including ethical considerations, is a part of the nurse anaesthetist (NA) education. Perioperative nursing care encompasses the dialogue and interaction with the patient as well as practical and technical procedures (Lindwall & von Post, 2008). The nature of perioperative care is complex and performed in a unique, high technology environment that may aggravate the patient–nurse relationship. Furthermore, in a busy daily surgical unit, the time pressure may be a challenge to the nursing care. The NA student meets patients who are extremely vulnerable, as they have to let go of control. They literally put their lives in the hands of strangers and their dignity may be at stake. Accordingly, safeguarding patient dignity should be a paramount concern for all health professionals involved in patient care. NA students are looking at incidents in the operating theatre with fresh eyes; they have not yet adapted to the standards and culture ruling in the operating theatre. Knowledge about student perception and interpretation of surgical patients' dignity is, therefore, appreciated and may contribute towards highlighting the patients' sense of vulnerability, feelings and needs. This may, in turn, be an incentive to develop consciousness and readiness of action not only among NA students but also among all health providers in clinical encounters where patient dignity is at stake.

## 2 BACKGROUND

To understand patient vulnerability and frailness, all professionals need to approach patients with discretion and carefully confirm human dignity (Eriksson, 1994). Eriksson (1994) describes human dignity as the profound concept of ethics in man. Human dignity can be expressed either as absolute or relative. Absolute dignity is given to humans at the beginning of time and involves the right to be confirmed as unique (Eriksson, 1995). Relative dignity is contextual—it can be broken down and violated or recreated and preserved depending on the situation (Edlund, Lindwall, von Post, & Lindström, 2013; Eriksson, 1995, 2006). According to Edlund, relative dignity is influenced by the world, the culture and the society that the person meets (Edlund, 2002; Edlund et al., 2013). Dignity is an ethical dimension and is expressed and reflected by health professionals through their virtues and attitude towards caring (Nåden & Eriksson, 2004).

Respecting human dignity is in accord with the International Code of Ethics for Nurses: “Inherent in nursing is a respect for human rights, including (…) the right to dignity and to be treated with respect” (International Council of Nurses, 2012). Ethical competence demands not only awareness and sensitivity but also moral judgement skills and willingness to do good.

Research has shown that operating theatre students have experienced both preserved and violated dignity during their clinical practice (Blomberg, Willassen, von Post, & Lindwall, 2015; Willassen, Blomberg, von Post, & Lindwall, 2015). Another study found that nurses working in pre‐hospital settings preserved patient dignity by attending to patients' needs. Furthermore, the nurses shielded the patient from other's gaze. Violated dignity was identified as disrespect and neglect (Abelsson & Lindwall, 2015). A study by Lindwall showed that the perioperative nursing care sometimes may be directed towards productivity at the expense of ethical considerations (Lindwall, von Post, & Eriksson, 2007).

To our knowledge, there are no studies on NA student experiences of how health professionals deal with patient dignity in perioperative practice. Accordingly, the aim of this study was to describe what NA students experience and interpret as being preserved and violated dignity in their clinical practice.

## 3 THE STUDY

### 3.1 Design

Based on the objective of the study, a hermeneutical approach inspired by Gadamer's (1989) understanding and interpretation was chosen. Gadamer focuses on the concept of pre‐understanding and fusion of horizons and emphasizes that those who express themselves and those who understand are connected by a common human consciousness that makes understanding possible.

### 3.2 Sample/Participants

The participants were 23 NA students partaking in their clinical practice at five hospitals in the eastern part of Norway. The students were between 26–40 years of age with more than 2 years of nursing experience. Seven students were male. As part of their education programme, they collected the data during their first perioperative clinical practice. The first period was chosen to avoid a possible adaption to the operating theatre culture, which may at a subsequent point influence their behaviour and attitudes. All students had beforehand participated in lectures on ethical issues, including the concept of patient dignity in perioperative practice. The goal of the lecture was to educate and prepare the students to focus on issues that they might encounter during their first perioperative practice period, when student attention is typically limited towards technical procedures and skills.

### 3.3 Data collection

Data were collected by the critical incident technique (Flanagan, 1958), which is a self‐reporting method focusing on incidents experienced by the participants. Flanagan (Flanagan, 1958, p. 335) contends that, “It should be emphasized that (…) the critical incident technique does not consist of a single rigid set of rules governing such (…) data collecting. Rather it should be thought of as a flexible set of principles which must be modified and adapted to meet the specific situation at hand” (p.335). Direct observations refer to incidents that participants witness or have been a part of and have influenced their emotions positively or negatively.

During 1 month in the Autumn of 2015, the students wrote down positive and/or negative critical incidents concerning dignity from perioperative practice by using a self‐constructed “Dignity critical incident form” designed by the authors. The form included questions such as How did the incident start? How did it develop? What did you think and feel and how did you act? The students reported a total of 20 observations on incidents of preserved dignity and 15 on violated dignity. All participating students gave their written consent and received beforehand orally and written information about the purpose and the methods of the study.

### 3.4 Hermeneutic text interpretation

The reported critical incidents were gathered into one text. To understand the text, the researchers were inspired by hermeneutic text interpretation. Gadamer (1989) highlights the meaning of language for creating the world where reality can be interpreted. The text should be understood and not become another's intention. The understanding of the text is based on the reader's existential and professional pre‐understanding (von Post & Eriksson, 1999). According to Gadamer (1989), all people have an existential pre‐understanding of life. Professional pre‐understanding is the result of one's professional education and experience as a nurse. The authors' pre‐understandings consist of the caring science perspective, medical knowledge, values, prejudices and ethical understanding as well as our experiences as nurse anaesthetists.

The text interpretation was done in five steps (Lindwall, von Post, & Eriksson, 2010).

#### 3.4.1 The first reading—integrating the text with the reader

The critical examination focuses on a text as an original source and its validity is found in its relevance to reality. The first reading began as an open reading and the text was read from the beginning to end without interruptions. During the reading, the reader asked the text questions. The text replied and posed statements such as: “Yes, this is perioperative practice”. The text addressed us as professional nurses (Gadamer, 1989).

#### 3.4.2 The second reading—fusion of horizons

Gadamer (1989) states that the dialogue with the text leads to a fusion of horizons; the reality of the text becomes a part of the reader. In the fusion of horizons, it became apparent that student experiences of patient dignity in a perioperative practice were complex and the approach to analysis was driven by the question: “Is this what the students experienced?”

#### 3.4.3 The third reading—new questions to the text

The following question was generated from the text: “How do NA students experience dignity in a perioperative practice?” The text was carefully read to discover significant expressions, quotations with common and distinguishing qualities.

#### 3.4.4 The fourth reading—summarizing main and subthemes

The text with the quotations was carefully read, in search of common features. The common features were categorized into two main themes and the distinctive qualities resulted in six subthemes. Each subtheme was described using quotes from the text.

#### 3.4.5 The fifth reading—a new understanding

The whole text was read again to reconfirm all themes compared with the whole text in search for a new understanding of the whole, from its parts and the parts from the whole, which Gadamer (1989) describes as the hermeneutic circle. This process of understanding involved an abstraction of the main themes and the subthemes formed a new understanding, a coherent whole that was considered valid and free from inner contradictions. According to Koskinen and Lindström (2013), hermeneutic reading is a working method where the researcher “takes a stance towards the text”. The five‐step text interpretation was at first done independently by the researchers. They then met during the process discussing the interpretations, and at a final meeting, consensus between the researchers was achieved.

### 3.5 Ethical considerations

The study was performed in accordance with the Declaration of Helsinki (Helsinki, 2013) which protects the research participants' anonymities, integrity and maintains public confidentiality. The project was approved by the local University College and the Norwegian Data Protection Authority.

## 4 RESULTS

The results show what NA students experienced and interpreted as incidents related to patient dignity in their perioperative practice. The common features were categorized into two main themes and the distinctive qualities resulted in six subthemes. Each subtheme will be described by using quotes from the text.

### 4.1 Preserving patient dignity

The main theme, preserving patient dignity, is demonstrated by the three following subthemes: “Allocating time for the patient”, “Inviting the patient to participate” and “Shielding the patient's body.”

#### 4.1.1 Allocating time for the patient

The NA students experienced that patient dignity was preserved when the health professionals allocated time by slowing down their work, listened and talked to the patient. One nurse dedicated time when encountering a young girl with a rare syndrome. He took a professional stance and addressed her respectfully as equal, without knowing whether she had a cognitive impairment:

I spent time to create confidence in the situation, as I realized that this girl had previously experienced a lot of negative encounters. She allowed me to hold her hand during induction of anaesthesia, which I felt was a vote of confidence from her (#34)

The nurse slowed down her work, acknowledging the patient's anxiety:

The patient was hiding under the blanket and her hands were shivering. The nurse sat down at the bedside and took the patient's hand, showing her empathy. The nurse took time to listen and recognized her fear, saying; you look a bit scared, poor thing (#26).

A patient arriving at the operating theatre seemed anxious and had several questions. The nurse allocated time to ease the patient's anxiety by chatting about everyday life:

We made efforts to reassure her, answering questions about monitoring and medication by giving her the answers she needed without scaring her by saying too much (#24).

The nurse used humour to reduce the patient's anxiety:

The nurse recognized the patient's anxious state of mind; she took her time and asked further questions about the patient's fear. Moreover, the nurse used humour to downplay the situation, not at the expense of the patient of course, but about herself and daily life experiences (#26).

By allocating time, the nurse alleviates patient suffering, and reduces anxiety and fear associated with surgery and anaesthesia. The NA student understood allocating time as taking responsibility for the patient's dignity and well‐being in a busy perioperative environment.

#### 4.1.2 Inviting the patient to participate

The students experienced that nurses informed the patients to prepare them for pending procedures. The patients were encouraged to take part in decision‐making related to their treatment. The nurses were responsive and listened carefully and thoroughly to the patients' needs and wishes. The patients were informed about different options and encouraged to make decisions themselves:

The nurse informed the patient that she needed to clean his skin with antiseptics before surgery and she asked him whether he preferred to be awake or asleep while she was cleaning (#16).

Patients were involved by sharing their story:

The nurse anaesthetist employed various communication methods to collect data and gain knowledge about how much the patient actually understood regarding her situation (#25).

The anaesthesiologist did not succeed in performing a spinal block and he explained respectfully that general anaesthesia might be another option:

Despite the information given, the patient was determined in her choice. The doctor said that he might give the spinal block another try […]. The patient looked relieved, which she also expressed verbally (#20).

The nurse invited the patient to participate in the induction of anaesthesia:

The patient was offered to hold the oxygen mask to promote empowerment and increase patient control of the situation (#26).

The health professionals preserved patient dignity by inviting the patients to participate in the perioperative nursing process. The health professionals provided sufficient, but not too much information to meet patients' needs and enabled the patients to take part in decision‐making.

#### 4.1.3 Shielding patient's body

The NA students experienced that patient dignity was preserved by not exposing patient bodies. The nurses covered sensitive areas during the perioperative procedures:

A shy girl, 12 years old, did not want to expose her upper body.

When the patient was anaesthetized, the nurse continued to treat the patient as if she actually was awake. Although it might have been convenient and time saving, the patient's upper body was never exposed (#22).

The patient was asked to lie down on the operating table and take off her shirt:

Before the patient stripped off her shirt, the operating theatre doors were closed. A nurse stood ready with a warm blanket to cover the patient […]. The patient was never uncovered during anaesthesia (#18).

The patient arrived at the operating theatre in his bed and was greeted by the operating theatre nurse and the nurse anaesthetist:

The patient got a warm blanket on top of the quilt, and subsequently, the quilt was carefully removed from under the blanket. The patient moved over to the operating table while covered with the blanket (#29).

The NA student experienced how nurses treated the patient as a unique human being, not as an object, they took responsibility for the patient's dignity by shielding the patient's body, thus protecting them from the cold and all eyes.

### 4.2 Violating patient dignity

The main theme, violating patient dignity, is demonstrated by the three following subthemes: “Alienation—ignoring the patient”, “Backbiting the patient” and “Invasion of the body's intimate sphere.”

#### 4.2.1 Alienation—ignoring the patient

The NA students experienced that the nurses ignored patient integrity. Nurses talked among themselves, they did not pay attention to the patients and they disregarded their wishes and worries:

The patient asked the nurse to remove the urinary catheter after surgery. Another nurse interfered and the two nurses were standing bedside discussing whether they should remove the urinary catheter. The patient disliked that the nurses had a conversation as if she were not there and she told them to stop talking. They seemed not to listen and continued (#9).

The NA students experienced how the surgeon entered the operating theatre talking loudly about the patient's health, seemingly not aware that the patient was awake:

He talked loudly about irrelevant and personal matters. Other health professionals carefully told him that the patient was awake. He replied, however, that he was perfectly aware of that, but it did not get any better. For example, he was, on request and still while the patient was awake, informed about the patient's blood pressure, whereon he burst out: “Isn't that an extremely high pressure”? (#11)

The student experienced how the nurse did not recognize the patient's anxiety, and the patient started to cry just before anaesthesia induction. The nurse ignored her and pressed the infusion pump start button:

After induction, I expressed my concerns about the patient. The nurse commented that the patient actually was an adult and that she (the nurse) would be more considerate if a younger patient was crying. The talk among the health professionals then continued condescendingly about the patient's problems (#13).

The NA student wanted to spend some time to reassure a patient that was extremely anxious and crying. However, the nurse interrupted the conversation:

I felt that I was interrupted during my attempts to gain the patient's trust and make him feel secure, as the nurse cut in: “Everything's going fine, let's start”. She then started the induction of anaesthesia (#13).

The NA students experienced how health professionals violated patient dignity by alienation—by not acknowledging the patient.

#### 4.2.2 Backbiting the patient

The NA students experienced how health professionals made malicious statements about the patient. Patients were subjected to condescending remarks, even when awake.

The patient was awake as the surgeon commented on the patient's physical and psychological status:

Here's a lot of fat, such a heavy leg! He is definitely not a marathon runner. Also, while the patient was emerging from sedation, the surgeon yelled: Does the patient have any history of dementia? (#4)

A skin graft failure had resulted in a large wound on the patient's upper arm. A surgeon entered the operating theatre talking very inappropriately and disrespectfully:

You need to be cautious about such ladies. If the two of you went dancing and performed a vigorous leap, her arm might fall off. He then laughed and left the room (#6).

The health professionals gave unnecessary remarks and had a disrespectful discussion on the patient's condition:

A big issue was made of the patient's bulimia. I felt that the patient was already stigmatized during the physicians' morning meeting (#27).

The NA students experienced how health professionals violated patient dignity by giving malicious and disrespectful remarks about the patients.

#### 4.2.3 Invasion of the body's intimate sphere

The NA students experienced how health professionals violated and exposed the body's intimate sphere during the perioperative process. The body was not properly covered and intimate areas were unnecessarily exposed:

A female adolescent, with slightly overweight and heavy breasts, was undressed and put in a very vulnerable position on the operation table. The patient, wearing knickers only, was positioned at the operating table in a hands and knees position. There were many persons in the operating theatre and the door was not closed. I could tell by the look in her eyes that she felt uncomfortable (#15).

Another NA student experienced that the patient was treated like an object; the nurse used an elderly male patient as a “table”, seemingly without considering any possible reaction from the awake patient:

The equipment was placed on the patient's abdomen, and every time, the nurse picked up equipment, such as vein catheter or fixation tape, she touched his genital area (#5).

The NA students experienced how health professionals violated the patient's body intimate sphere by exposing the patient's body and not shielding them from all eyes. Thus, the patients were afflicted with unnecessary sufferings.

### 4.3 New understanding

**Figure 1:**
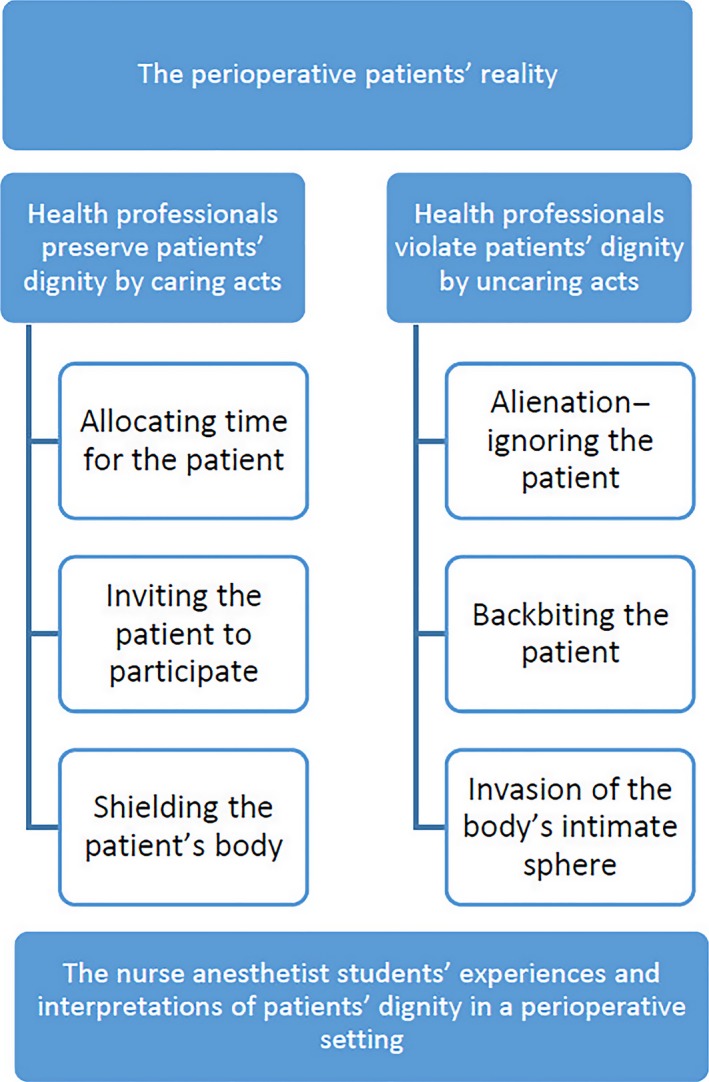
The nurse anaesthetist students' experiences and interpretations of patients' dignity in a perioperative setting

In accordance with Gadamer (1989), the present findings led to new understanding of how students experience and interpret patient dignity in perioperative practice in their first perioperative practice period before the operating theatre culture had become a part of them and constructed a new reality that may influence their behaviour and attitude (Figure 1). Preserving patient dignity can be understood as a caring act and violating dignity as an uncaring act (Lindstrøm, Lindholm, & Zetterlund, 2010).

The new understanding of how NA students experienced patient dignity can be summarized into the following presumptions:

- Allocating time and communicating implies allowing the patients to express their distress, anxiety and concerns, and preparing them for pending procedures.
- Inviting the patients to participate implies that the patients feel reassurance, trust and control of the situation.
- Shielding patient bodies implies that the patients get a feeling of well‐being and that they are treated as human beings.

- Alienating and ignoring the patients implies that patient needs and preferences are ignored.
- Backbiting implies disrespect, allowing depreciation of the patient integrity.
- Invasion of the body's intimate sphere implies that the patients may feel objectified and demeaned.

Learning a profession relies on approaches not only theoretical but also clinical. Thus, positive role models are essential to provide the NA students different tools; how to perform with delicacy and discretion in different situations (Grob, Leng, & Gallagher, 2012). The experiences in this study may be an incentive to further reflection to develop awareness and take a stance of this important issue. In addition, the findings of this study could serve as a basis for interventions on how to promote an ethical and dignified culture and how to deal with undignified care in perioperative practice.

## 5 DISCUSSION

This study describes that NA students experienced that patient dignity was preserved when health professionals allocated time, invited them to participate and shielded their bodies. Patient dignity was violated when health professionals alienated and ignored them, backbit them and invaded their intimate sphere.

Patients undergoing anaesthesia and surgery are particularly vulnerable. Preserving patient dignity should be of concern to all health professionals and is in line with the ICN Code of Ethics for Nurses (International Council of Nurses, 2012). The NA students experienced that health professionals in many situations contributed to preservation of patient dignity.

Allocating time for health professionals to accommodate trust and confidence is demonstrated by slowing down their work and listening and talking to the patients. Allocating time is a way for the health professional to be courteous, helpful and considerate, which may give the patients a feeling of being valued and being in control (Baillie, 2009; Baillie & Ilott, 2010).

Allocating time may be challenging in a busy, daily work routine. However, a small amount of time spent to console patient dignity and alleviate distress is necessary to fulfil the ethical obligations of health professionals.

Health professionals should act in a way that helps patients feel comfortable, in control and valued. This study shows that patients were involved in time point of procedure, choice of anaesthetic technique and administration of oxygen. Thus, the patients may have felt comfortable, in control and valued. These findings are also demonstrated in Baillie's research as core elements in preserving patient dignity (Baillie, 2009). Another study by Forsberg found that patients appreciated the opportunity to participate in decisions about their care (Forsberg, Vikman, Wälivaara, & Engström, 2015). Furthermore, the patients should receive sufficient information. However, the information should not be too detailed as it may cause distress and anxiety. This requires sensitivity from the health professionals towards the patient (Ekman et al., 2011). Reassurance may be a technique to reduce anxiety and distress. When reassurance is not provided, patients may feel ignored and their dignity violated.

The findings show that the NA students regarded patient privacy as an important issue and observed that the health professionals shielded patient bodies. They treated the patient with respect regardless of whether the patient was fully awake or not. This is in line with the findings of Blomberg et al. (2015). Health professionals should be sensitive and discreet towards the patient's need for privacy and regard the patient as a person and not an object (Gallagher, 2011). Furthermore, maintaining privacy contributes to patient satisfaction (Forsberg et al., 2015).

The high technological environment in the operating theatre has the potential to distance the health professionals from the patients and jeopardize the quality of care (Bull & FitzGerald, 2006). Undergoing surgery and anaesthesia implies that the patients relinquish their bodies to alien health professionals. In addition, the environment in the operating theatre may be perceived as unfamiliar and inhospitable, and patients are susceptive to be defenceless and vulnerable. The NA students experienced that health professionals contributed to violation of patient dignity in terms of alienation, backbiting and invasion of patient intimate sphere.

The NA students observed that patients were alienated when ignored and not paid attention to. This is in accordance with a study by Willassen et al. (2015), where operating theatre nurse students observed that health professionals rendered the patients as being invisible. A non‐responsive manner is perceived by the patients as inhumane and unkind and violates their dignity (Hankela & Kiikkala, 1996). In a study by Forsberg et al. (2015), patients suggested perioperative care improvement by health professionals allocating time and being responsive.

The encounter between patients and the health professional in the operating theatre is time limited. A hurried way of action may demonstrate that the health professional is unengaged and unpleased (Forsberg et al., 2015). However, the caring acts may sometimes be pushed aside for the benefit of anaesthesia and surgery procedures.

The NA students experienced negative remarks about patient physical and psychological status. This is also confirmed by Willassen et al. (2015). Health professionals should be extremely considerate and attentive when talking, as patients may perceive verbal conversation even when anaesthetized (Cook et al., 2014). Verbal abuse, making jokes at patients' expense and putting them down may lead to negative attitudes, resulting in a harmed, hurt or demeaned patients. The negative attitudes may also influence the attitudes towards patients in general and have a negative impact on the perioperative environmental culture.

Health professionals sometimes are susceptible to perceive the patients as an object (Smith & Mishra, 2010). The NA students observed that patient bodies were unshielded and exposed. This was also found in a former study where operating nurse students reported that the patients were objectified (Willassen et al., 2015). A study of intensive care showed that 40% of patients had intimate areas exposed (Turnock & Kelleher, 2001). Lack of compassion without the consideration of patient feelings and without acknowledging patient vulnerability and dignity is a violation of the caring act (Wiklund Gustin & Wagner, 2013). Maintaining privacy is a significant issue for patients and the importance of this issue is underlined in a systematic review (Rhodes, Miles, & Pearson, 2006).

Lèvinas (1989) contends that ethics should be characterized as a person's consciousness about other's suffering. A relation is created through a person's awareness and notion of the other's vulnerability. The present study creates new questions in terms of ethics and value conflicts in perioperative practice and the view of humanity is of immense importance in the encounter between health professionals and patients in this setting.

Most patients are vulnerable when in need of anaesthesia and surgery. The environment is unknown for the patients and several different health professionals are present as the patients enter the operating theatre. In some instances, the health professionals' focus may shift from patient to procedures. The health professionals are committed to focus on the caring act as well as the “doing” to avoid being instrumental and distanced from the patients caring demand. The danger of acting in a time‐saving manner and value quantity above quality is labelled as “fast‐ethics”. It is, however, suggested that health professionals should use “slow ethics” to avoid practice that compromises the patient integrity and the professional ethics (Ann Gallagher, 2013). Gallagher states that dignity should be explored as an another‐regarding as well as a self‐regarding value. As central human aspects, Gallagher emphasizes vulnerability and fallibility as potential risk factors for indignity and humiliation. By observing and taking part in acts that violate patient dignity, the health professionals also violate their own dignity and maintaining dignity is an important element of nursing care. The NA students expressed frustration and powerlessness. This is like another study where the nurses expressed anger and shame to be a part of a team that compromised patient dignity (Walsh & Kowanko, 2002). When NA students experience that patient dignity is violated, they end up unwittingly in situations causing inner value conflicts that they should not expect or may not be prepared for (von Post, 1998).

Asking NA students to observe and report their experiences in their first clinical practice not only made them aware of means to preserve patient dignity but also how “easy” it is to violate patient dignity. The main purpose was to draw attention to promoting dignity in clinical practice, by observing, reflecting, knowing and doing better, and improving their ethical competence when facing vulnerability and humiliation. Concentrating on their own lived experience may be a means of promoting dignity and to improve nursing practice. Students are vulnerable as they interact and treat patients under constant supervision and evaluation. The perioperative care takes place in a hierarchic culture (Lindwall & von Post, 2008) and this environment demands courage to call out and address unethical practice.

### 5.1 Limitations

The study is based on limited number of participants and NA students' interpretation of the situation. One could question whether the patients actually felt that their dignity was preserved or violated, but it should be noted that the students are all experienced nurses from other fields of nursing.

## 6 CONCLUSION

Overall, the NA students' experiences of violation of patient dignity is very similar to experiences reported by others (Abelsson & Lindwall, 2015; Lindwall & von Post, 2014; Turnock & Kelleher, 2001), including experiences of operating theatre nurse students (Willassen et al., 2015). The fact that both the present study and the study by Willassen et al. (2015) were performed in the same environmental practice, albeit in different hospitals, underlines the need for strengthening the ethical awareness in perioperative care. NA students should develop sensitivity to how their interactions with patients may affect patient dignity to sustain and improve their ethical competence. There may also be a need to strengthen their competence to deal with situations of undignified care. NA students observing violation of patient dignity described a feeling of unease. The NA students expected a preservation of patient dignity, and a value conflict may cause the students to suffer and gradually demotes job satisfaction and working moral.

Learning a profession relies on approaches not only theoretically but also clinically. Thus, positive role models are essential to provide the NA students different tools; how to perform with delicacy and discretion in different situations (Grob et al., 2012). The experiences in this study may be an incentive to further reflection for developing a stance and awareness of this important issue. Furthermore, the findings of this study could serve as a basis for interventions on how to promote an ethical and dignified culture and how to deal with undignified care in perioperative practice.

## ACKNOWLEDGEMENTS

The authors thank all the students for their participation in the study and for sharing their critical incidents and making it possible to carry out this study.

## CONFLICT OF INTEREST

The authors declare that they do not have any conflicts of interest.

## AUTHORS CONTRIBUTIONS

All authors meet the authorship criteria. They all had access to the study data, contributed to the analyses and interpretation of the data. Each one has critically reviewed each draft, provided comments and guidance to the manuscript, and approved the final version.
